# Excision of Mucocele Using Diode Laser in Lower Lip

**DOI:** 10.1155/2016/1746316

**Published:** 2016-12-21

**Authors:** Subramaniam Ramkumar, Lakshmi Ramkumar, Narasimhan Malathi, Ramalingam Suganya

**Affiliations:** ^1^Department of Oral & Maxillofacial Surgery, Faculty of Dental Sciences, Sri Ramachandra University, Chennai, India; ^2^Dr. Ram's Dental Care & Maxillofacial Center, Chennai, India; ^3^Department of Oral Pathology and Microbiology, Faculty of Dental Sciences, Sri Ramachandra University, Chennai, India

## Abstract

Mucoceles are nonneoplastic cystic lesions of major and minor salivary glands which result from the accumulation of mucus. These lesions are most commonly seen in children. Though usually these lesions can be treated by local surgical excision, in our case, to avoid intraoperative surgical complications like bleeding and edema and to enable better healing, excision was done using a diode laser in the wavelength of 940 nm.

## 1. Introduction

Mucoceles are known as “mucus filled cavities” usually present in the oral cavity, lacrimal sac, and paranasal sinuses [1]. Mucus extravasation and mucus retention are the two most frequently occurring primary mechanical obstructive diseases of salivary glands [2]. Formation of mucus extravasation cyst is mainly due to mechanical trauma causing rupture of ductal system of salivary gland and mucin spills into adjacent soft tissues [3, 4]. Mucus retention cyst is formed markedly by obstruction of salivary ductal walls causing dilatation of ducts without spillage of mucin [5, 6].

## 2. Case Report

**Figure 1:**
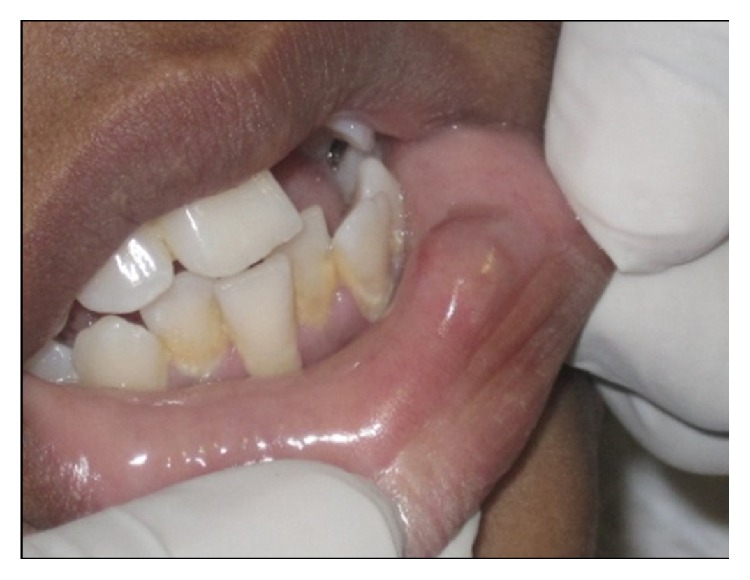
Swelling in the left labial mucosal region.

A 16-year-old female presented with a swelling in the lower left labial mucosal region for the past few months. She complained of intermittent swelling which often bursts and disappears for a few days. On clinical examination, lesion was soft, painless, fluid-filled, and approximately 1 × 1 cm in size (Figure 1). The history and clinical presentations were consistent with mucocele. Various treatment modalities such as surgical incision, cauterization, and laser excision were explained to the patient's guardian and obtained willingness to perform the most recent treatment option of laser excision.

**Figure 2:**
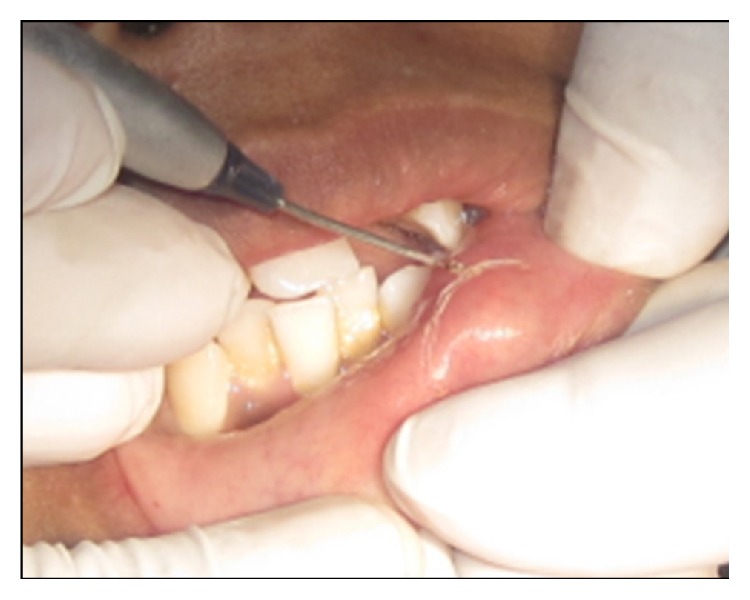
Application of laser: parameters, 940 nm and 1.5 W; continuous mode, 400 microns.

**Figure 3:**
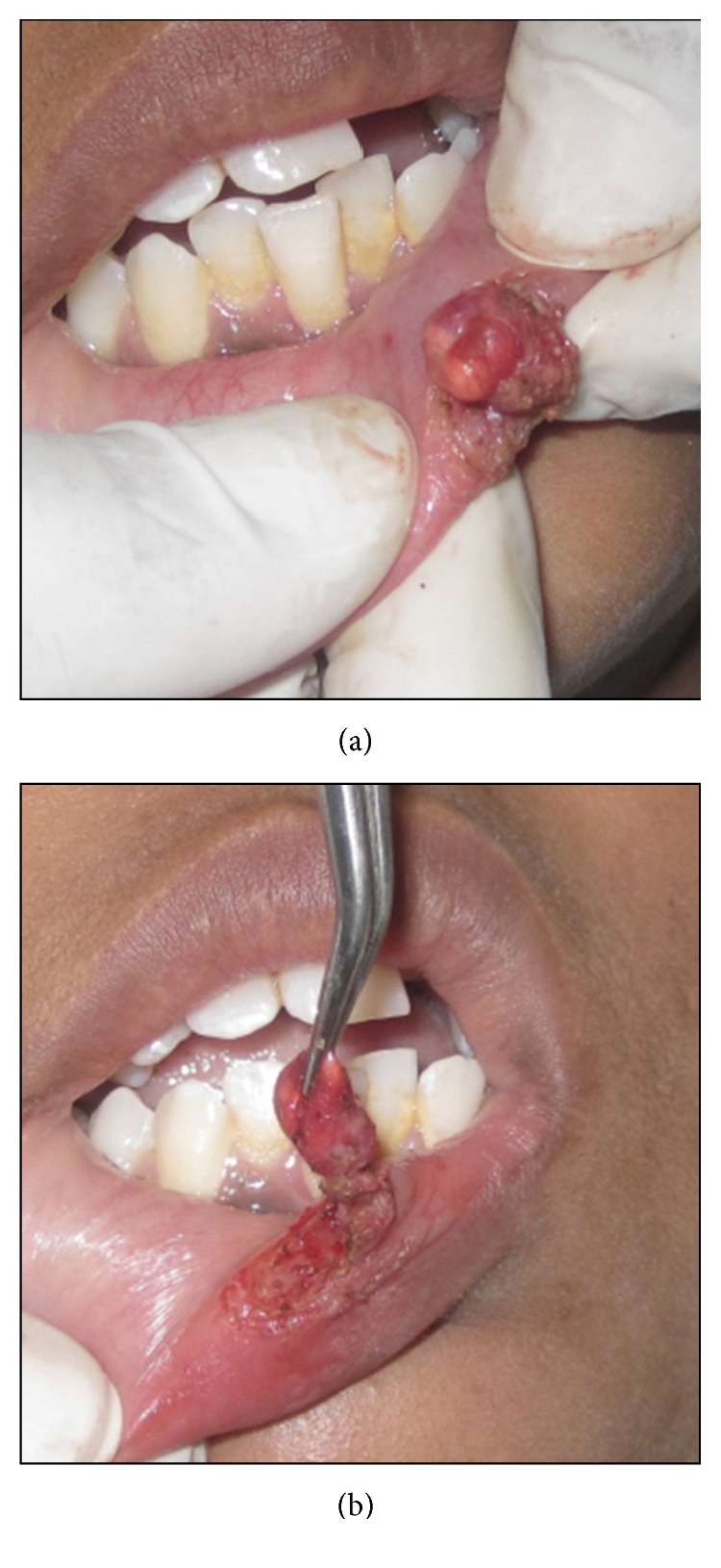
Intraoperative photograph.

**Figure 4:**
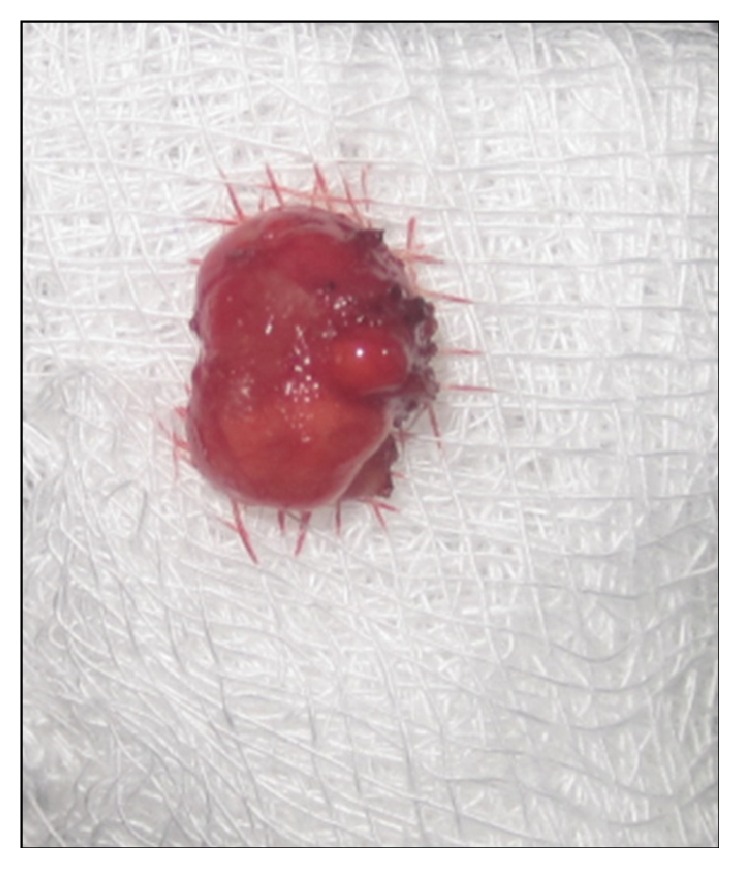
Excised specimen.

**Figure 5:**
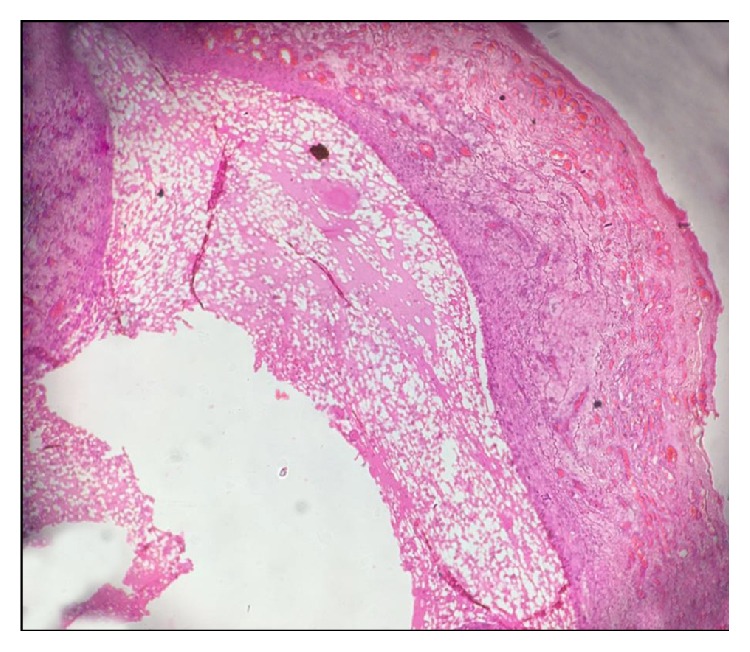
Photomicrograph showing H&E 40x view cystic cavity lined by thick fibrous capsule. Cystic lumen contains mucin, foamy macrophages, and chronic inflammatory cells.

**Figure 6:**
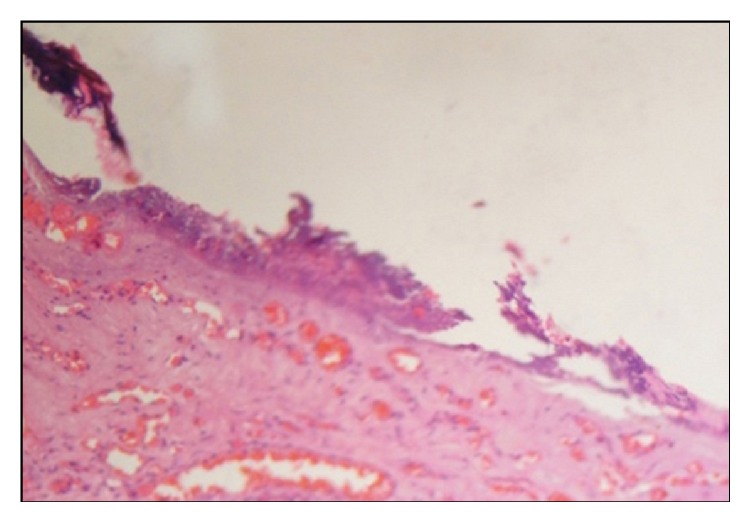
Photomicrograph showing H&E 40x view zone of coagulation necrosis surrounding the intended biopsy material.

**Figure 7:**
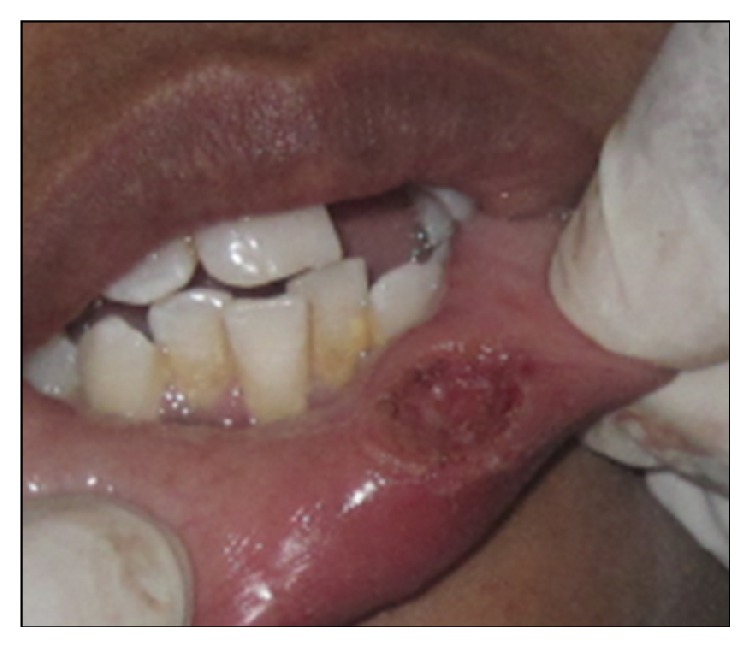
Photograph showing immediate postoperative day.

**Figure 8:**
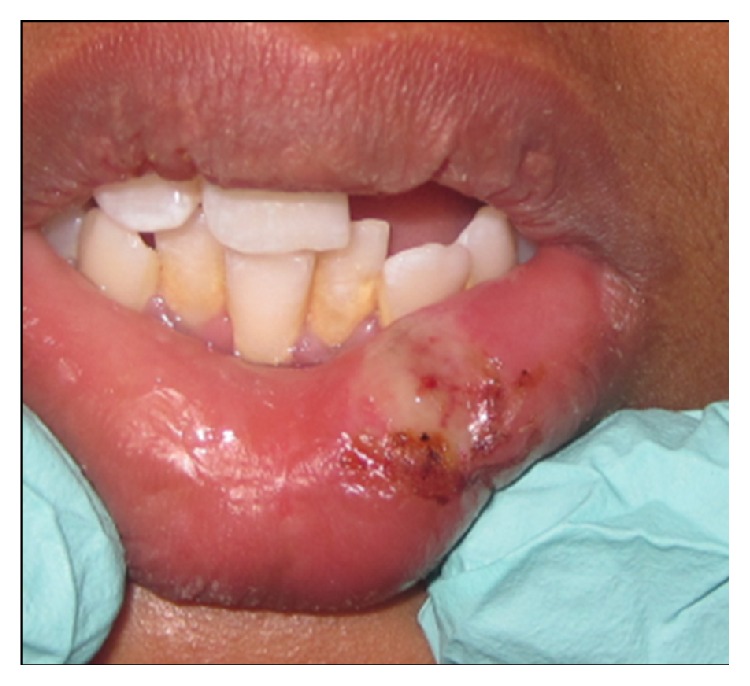
Photograph showing postoperative view: Day 1.

**Figure 9:**
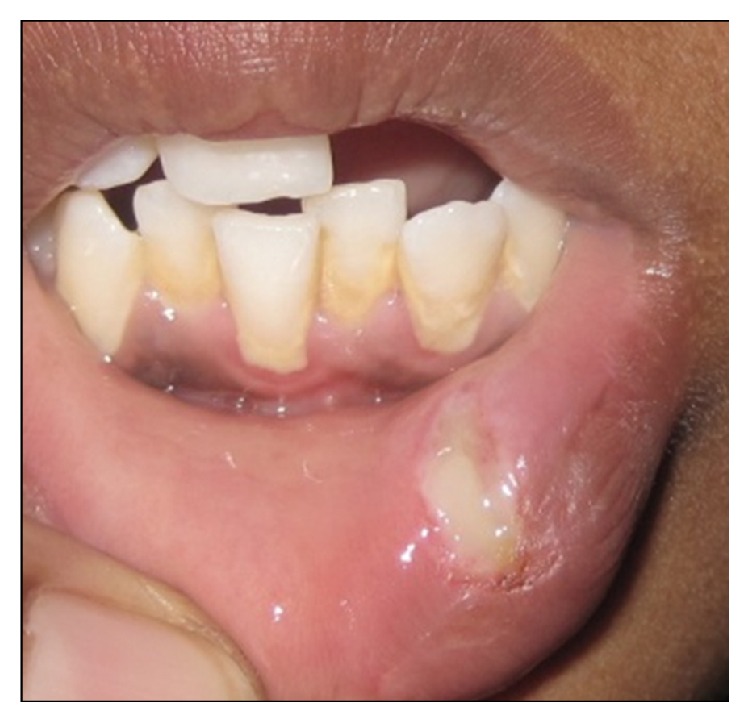
Photograph showing postoperative view: Day 4.

**Figure 10:**
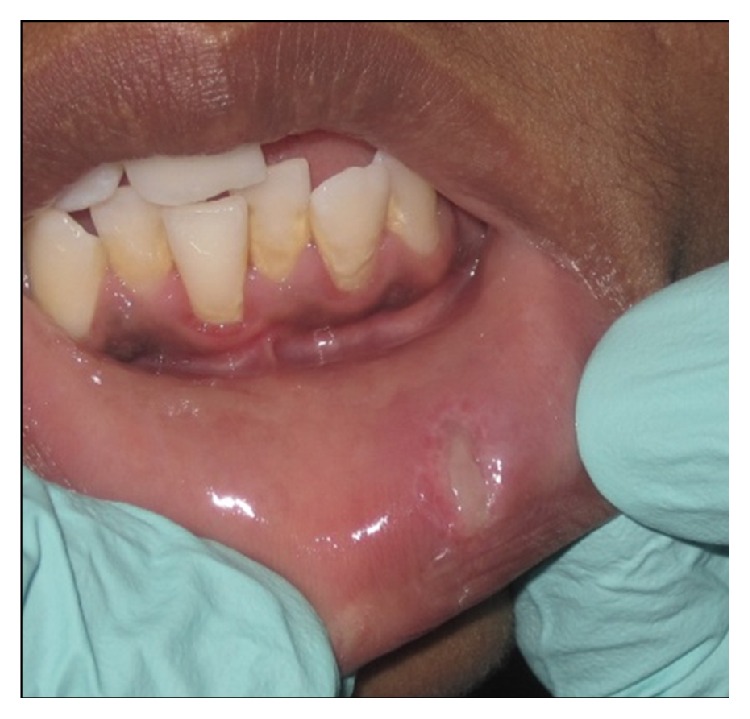
Photograph showing postoperative view: Day 8.

**Figure 11:**
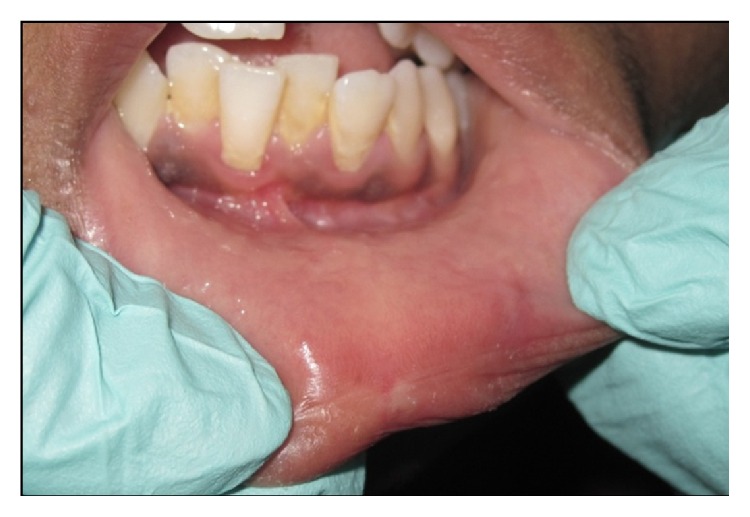
Photograph showing postoperative view: Day 45.

Following minimal infiltration of 1 : 2,00,000 Xylocaine, the lesion was excised using soft diode laser in wavelength of 940 nm, 400 *μ*m diameter tip at 1.5 W in continuous mode. The incision was placed on the uppermost site of the lesion and complete excision was performed (Figures 2, 3(a), and 3(b)). The specimen (Figure 4) was subjected to histopathological examination and showed cystic cavity lined by thick fibrous capsule. Cystic lumen contains mucin, foamy macrophages, and chronic inflammatory cells. Areas of coagulation necrosis surrounding the intended biopsy material were also evident. Adjacent mucous salivary gland was also seen. With all these histopathological features, diagnosis of mucous extravasation cyst was given (Figures 5 and 6). Patient was prescribed analgesics. There was uneventful healing on 45 days of follow-up (Figures 7, 8, 9, 10, and 11).

## 3. Discussion

Mucocele is the second most common lesion in the oral cavity followed by irritational fibroma. Incidence of this lesion occurs in the age group between 10 and 29 years with equal gender distribution [7]. Mucoceles appear as dome-shaped mucosal swellings with the characteristic accumulation of mucin. These lesions usually impart bluish, transparent hue of variable size from 1-2 mm to several centimetres in dimension [3, 8]. Lower lip is the most common site of occurrence of mucocele followed by the buccal mucosa and floor of mouth [9]. Depending upon the size and location of mucoceles, the various clinical features include external swelling and interference with mastication, swallowing, and speech and discomfort might occur [7]. Histopathologic examination of mucocele often reveals formation of well-circumscribed, cyst-like space surrounded by granulation tissue and the presence of mucinophages in the collapsed wall of granulation tissue [10]. The adjacent salivary gland tissue should also be present because mucocele should always be removed along with feeder glands/ducts which minimize recurrence of the lesion.

There are various treatment aspects available for the management of mucocele early: scalpel incision, complete surgical excision, marsupialization, micromarsupialization, intralesional injections of corticosteroids, cryosurgery, laser ablation, sclerosing agent, and electrocautery methods [8].

The main advantages of soft tissue laser applications are minimal intraoperative bleeding and swelling and postoperative pain and very less surgical time, scarring, and coagulation, without any need of suturing after excision because of natural wound dressing due to denatured proteins. Various procedures like minor and major soft tissue surgery, bone cutting, and implant exposure with bone removal can be performed in patients with bleeding disorders by using soft tissue lasers [11, 12].

The semiconductor diode lasers are available in different wavelengths such as 810–830 nm, 940 nm, and 980 nm [13]. The present case was performed by using 940 nm in which excellent hemostasis can be achieved due to good affinity for pigments like haemoglobin [14].

Diode lasers can be a useful alternative to larger surgical lasers such Er:YAG and CO2 lasers. Their small size and low cost are distinct advantages. They can give a well-defined cutting edge, as well as coagulation and hemostasis during excisions [12].

Absorption of laser energy into the target tissue releases heat by photothermal process which further causes intra- and extracellular vaporization of cells with resultant cellular explosion and tissue ablation. Adjacent lateral tissues will also absorb heat, on enough time of laser application. This will occur in concentric serial circles around the homogeneous target tissue. Reversible or irreversible damage of areas surrounding the target tissue by the thermal effects of laser results in zone of coagulation necrosis. Delayed healing and a larger wound site may occur on increased time of laser application. On the other hand, sealing of small diameter of vessels rather than the area of coagulation necrosis provides advantages like hemostasis during laser surgery. Area of adjacent coagulation ends with less bleeding at surgical site. The presence of border of necrotic and coagulated tissue in an incisional or excisional biopsy may result in intricacy of histopathological identification [15].

Histological examination of laser excised tissue shows improved epithelization and lesser inflammation. Intact basement membrane and connective tissue matrix can also be observed. Matrix proteins initiate reparative synthesis on these tissues. Resistance of matrix proteins against laser application and replacement as well as removal of residual matrix is responsible for reduced scarring and contraction [16].

## 4. Conclusion

Our present case report reveals knowledge about using diode laser for the treatment of mucocele with a variety of beneficial effects such as minimal anesthesia, less procedural timings, good surgical site visualization, hemostasis, and minimal carbonization in 45 days of periodical follow-up. Laser application makes it possible to reduce apprehension and fear in pediatric and geriatric patients.
